# From belongingness to reduced loneliness: the roles of participation intensity and self-esteem in ACG communities

**DOI:** 10.3389/fpsyg.2026.1712286

**Published:** 2026-05-05

**Authors:** Yixiao Lu

**Affiliations:** Department of Educational Psychology and Counseling, Faculty of Educational Studies, Universiti Putra Malaysia, Serdang, Malaysia

**Keywords:** ACG communities, belongingness, cross-sectional study, loneliness, mediation analysis, participation intensity, self-esteem

## Abstract

**Background:**

A sense of belonging is widely recognized as a fundamental human need and a key factor in psychological adjustment, particularly during adolescence and emerging adulthood. With the rapid development of digital media, online interest-based communities, such as anime, comic, and game (ACG) communities, have become important social environments where young individuals engage in social interaction, identity expression, and emotional exchange. However, the mechanisms through which perceived belongingness in such communities relates to loneliness remain insufficiently explored.

**Methods:**

This study employed a cross-sectional, questionnaire-based design to examine the relationships among perceived belongingness in ACG communities, participation intensity, self-esteem, and loneliness. A total of 300 adolescents and young adults participated in the study. Data were analyzed using correlation analysis and serial mediation modeling with bootstrapping procedures.

**Results:**

The results indicated that stronger perceived belongingness in ACG communities was significantly associated with lower levels of loneliness. Participation intensity and self-esteem both played significant mediating roles in this relationship. Specifically, belongingness was positively associated with participation intensity and self-esteem, which in turn were negatively related to loneliness, forming a significant serial mediation pathway.

**Conclusion:**

The findings suggest that ACG communities may function as meaningful social environments that support psychological wellbeing by enhancing individuals’ sense of belonging and self-worth. This study contributes to the literature by providing an integrated framework linking belongingness, behavioral engagement, and intrapersonal processes to loneliness among adolescents and young adults.

## 1 Introduction

Adolescence and emerging adulthood represent critical developmental stages during which individuals become increasingly sensitive to social relationships, identity formation, and emotional wellbeing. A substantial body of research has demonstrated that a sense of belonging is a fundamental human need and plays a central role in psychological adjustment. Individuals with a stronger sense of belonging tend to report higher levels of self-esteem and lower levels of loneliness, whereas a lack of belonging is associated with psychological distress and maladjustment (Baumeister and Leary, 1995; Hawkley and Cacioppo, 2010; Qualter et al., 2015). From this perspective, belongingness is not only a basic motivational force but also a key factor influencing emotional and social functioning.

With the rapid development of digital technologies, the ways in which young people experience social connection have undergone significant transformation. In addition to traditional offline contexts such as family and school, online environments have become increasingly important spaces for social interaction and identity construction. Online communities, particularly those formed around shared interests, provide opportunities for individuals to engage in communication, collaboration, and self-expression (Baym, 2018; Booth, 2015; Kraut et al., 2014; Verduyn et al., 2017). Among these, anime, comic, and game (ACG) communities have emerged as prominent subcultural spaces, especially among adolescents and young adults. These communities are characterized by shared symbolic systems, participatory practices, and strong group identification, offering members a sense of belonging and social connection that may not be readily available in offline settings (Ito et al., 2010; Jenkins, 2013; Hills, 2013).

Existing research on online and fandom communities suggests that participation in such environments can be associated with positive psychological outcomes, including enhanced social connectedness, identity affirmation, and emotional support. For instance, online interaction has been shown to contribute to individuals’ perceived social support and wellbeing, although its effects may vary depending on the nature and intensity of engagement (Burke et al., 2010; Kross et al., 2013; Valkenburg and Peter, 2011; Nesi et al., 2018). However, prior studies have often focused either on general online behavior or on potential risks such as excessive use and social withdrawal, while relatively fewer studies have systematically examined the underlying psychological mechanisms that link belongingness to loneliness within specific interest-based communities such as ACG contexts.

In particular, the processes through which perceived belongingness in ACG communities relates to loneliness remain insufficiently understood. Participation intensity may represent a behavioral pathway, reflecting the degree to which individuals actively engage in community-related activities, while self-esteem may function as an intrapersonal mechanism through which social experiences are internalized. According to sociometer theory, self-esteem reflects individuals’ perceptions of their social acceptance and relational value, suggesting that experiences of belonging may enhance self-esteem, which in turn reduces feelings of loneliness (Leary and Baumeister, 2000; Orth et al., 2012). At the same time, greater participation in community activities may reinforce social bonds and increase opportunities for meaningful interaction, thereby further contributing to psychological wellbeing (Kaye et al., 2021; Kowert and Oldmeadow, 2015).

Despite these theoretical considerations, few studies have integrated belongingness, participation intensity, self-esteem, and loneliness within a unified analytical framework in the context of ACG communities. Addressing this gap is important for advancing the understanding of how interest-based online communities influence psychological outcomes among youth. Therefore, the present study aims to examine the relationships among perceived belongingness in ACG communities, participation intensity, self-esteem, and loneliness among adolescents and young adults. It is proposed that stronger perceived belongingness is associated with lower levels of loneliness, both directly and indirectly through increased participation intensity and enhanced self-esteem. Specifically, belongingness is expected to be positively related to participation intensity and self-esteem, which in turn are negatively associated with loneliness, forming a potential serial mediation pathway.

## 2 Methods

### 2.1 Research design

The present study employed a cross-sectional, questionnaire-based research design to examine the relationships among ACG community belongingness, participation intensity, self-esteem, and loneliness. All variables were measured at a single time point using self-report instruments. Although cross-sectional designs do not permit causal inference, they are widely used to examine associations among psychological constructs and to explore potential mediation mechanisms in social and developmental research contexts. Accordingly, the findings of this study are interpreted within an associational framework.

### 2.2 Participants and procedure

Participants were Chinese adolescents and young adults with experience participating in anime, comic, and game (ACG)–related communities. Inclusion criteria required that participants (a) had engaged in ACG-related activities (e.g., anime viewing, gaming, fandom interaction, or content creation) and (b) had experience interacting with others within ACG-related community contexts, either online or offline.

Data were collected using an anonymous online questionnaire distributed through social media platforms, online forums, and ACG-related communities. A convenience sampling strategy was employed. Data collection took place between January 2025 and September 2025.

A total of 300 participants completed the questionnaire. Participants ranged in age from 13 to 24 years, with 55% identifying as male and 45% as female. Participants were drawn from multiple regions in China, including the Yangtze River Delta (Shanghai, Jiangsu, Zhejiang), Southwest China (Chengdu, Chongqing), and Western regions (Tibet and Xinjiang), reflecting a degree of geographic diversity.

All participants were informed of the study purpose and the voluntary nature of participation prior to completing the questionnaire. Informed consent was obtained from all participants, and for participants under the age of 18, consent was obtained from their legal guardians in accordance with ethical guidelines. Demographic characteristics of the participants are presented in Table 1.

**Table 1:** Demographic characteristics of participants (*N* = 300).

Age group	*n*	%
13–15	26	8.67
16–18	42	14.00
19–21	136	45.33
22–24	96	32.00

Values are presented as frequencies (*n*) and percentages (%) unless otherwise indicated.

### 2.3 Measures

All variables were assessed using self-report measures. Unless otherwise specified, responses were recorded on Likert-type scales, with higher scores indicating higher levels of the corresponding constructs.

Perceived belongingness in ACG communities was assessed using a modified self-report scale adapted from established measures of social connectedness and belongingness (Baumeister and Leary, 1995). The items captured participants’ subjective sense of acceptance, identification, and emotional attachment within ACG-related communities (e.g., “I feel that I belong to the ACG community”). Responses were rated on a 5-point Likert scale ranging from 1 (strongly disagree) to 5 (strongly agree), with higher scores indicating stronger perceived belongingness.

Participation intensity was measured using a self-developed Participation Intensity Scale consisting of seven items assessing the frequency and depth of engagement in ACG-related activities. Higher scores reflected greater levels of participation. The scale demonstrated excellent internal consistency (Cronbach’s *α* = 0.92). Exploratory factor analysis supported a single-factor structure, with satisfactory sampling adequacy (KMO = 0.93) and significant Bartlett’s test of sphericity, indicating the suitability of the data for factor analysis.

Loneliness was assessed using a standardized self-report measure capturing perceived social isolation and dissatisfaction with interpersonal relationships. Higher scores indicated higher levels of loneliness (Hawkley and Cacioppo, 2010).

Self-esteem was measured using a widely used global self-report scale assessing individuals’ overall evaluation of self-worth. Higher scores indicated higher levels of self-esteem, consistent with sociometer theory perspectives on self-evaluation (Leary and Baumeister, 2000; Orth et al., 2012).

### 2.4 Control variables

Age and gender were included as control variables in all analyses. Age was categorized into four groups (13–15, 16–18, 19–21, and 22–24), and gender was coded as a binary variable. These variables were controlled based on prior research indicating their associations with psychological adjustment indicators such as loneliness and self-esteem (Qualter et al., 2015; Orth et al., 2012).

### 2.5 Data analysis

Data analyses were conducted using IBM SPSS Statistics (version 27.0.1). Descriptive statistics and reliability analyses were first calculated for all study variables. Pearson correlation analyses were then conducted to examine bivariate relationships among belongingness, participation intensity, self-esteem, and loneliness.

To further examine the relationships among variables, regression-based analyses were performed. In addition, a serial mediation model was tested using the PROCESS macro (Model 6) with 5,000 bootstrap samples to examine whether participation intensity and self-esteem functioned as sequential mediators in the association between belongingness and loneliness (Hayes, 2022). Indirect effects were considered significant when the 95% bias-corrected confidence intervals did not include zero (Preacher and Hayes, 2008).

## 3 Results

### 3.1 Descriptive statistics and correlations

Descriptive statistics and Pearson correlation coefficients for all study variables are presented in Table 2.

**Table 2:** Means, standard deviations, and correlations among study variables.

Variable	M	SD	1	2	3	4
Belongingness	3.25	0.92	—			
Participation intensity	3.48	0.99	0.41***	—		
Loneliness	2.63	0.91	−0.39***	−0.34***	—	
Self-esteem	3.37	0.97	0.36***	0.30***	−0.41***	—

M, mean; SD, standard deviation. ***p < 0.001.

Participants reported moderate levels of ACG community belongingness (M = 3.25, SD = 0.92) and participation intensity (M = 3.48, SD = 0.99). The mean level of loneliness was relatively low (M = 2.63, SD = 0.91), whereas self-esteem was moderately high (M = 3.37, SD = 0.97). All variables were approximately normally distributed, and no substantial ceiling or floor effects were observed.

Pearson correlation analyses revealed significant associations among all key variables. Belongingness was positively correlated with participation intensity (*r* = 0.41, *p* < 0.001) and self-esteem (*r* = 0.36, *p* < 0.001), and negatively correlated with loneliness (*r* = −0.39, *p* < 0.001). Participation intensity was negatively associated with loneliness (*r* = −0.34, *p* < 0.001) and positively associated with self-esteem (*r* = 0.30, *p* < 0.001). In addition, loneliness was negatively correlated with self-esteem (*r* = −0.41, p < 0.001). These results provided preliminary support for the hypothesized relationships.

### 3.2 Mediation analysis

A serial mediation analysis was conducted using PROCESS Model 6 with 5,000 bootstrap samples to examine whether participation intensity and self-esteem sequentially mediated the relationship between belongingness and loneliness.

The results of the mediation analysis are presented in Table 3.

**Table 3:** Results of the serial mediation analysis.

Part A. Regression coefficients					
Outcome variable	Predictor	*B*	SE	*t*	*p*
Participation intensity	Belongingness	0.52	0.04	12.87	<0.001
Self-esteem	Participation intensity	0.34	0.05	6.80	<0.001
Loneliness	Self-esteem	−0.41	0.05	−8.20	<0.001
Loneliness	Belongingness (direct)	−0.23	0.04	−5.75	<0.001

Part B. Indirect effects (bootstrap = 5,000)			
Indirect path	Effect	SE	95% CI
Belongingness → participation → loneliness	−0.054	0.023	[−0.100, −0.011]
Belongingness → self-esteem → loneliness	−0.065	0.020	[−0.107, −0.031]
Belongingness → participation → self-esteem → loneliness	−0.015	0.007	[−0.030, −0.004]

**Figure 1:**
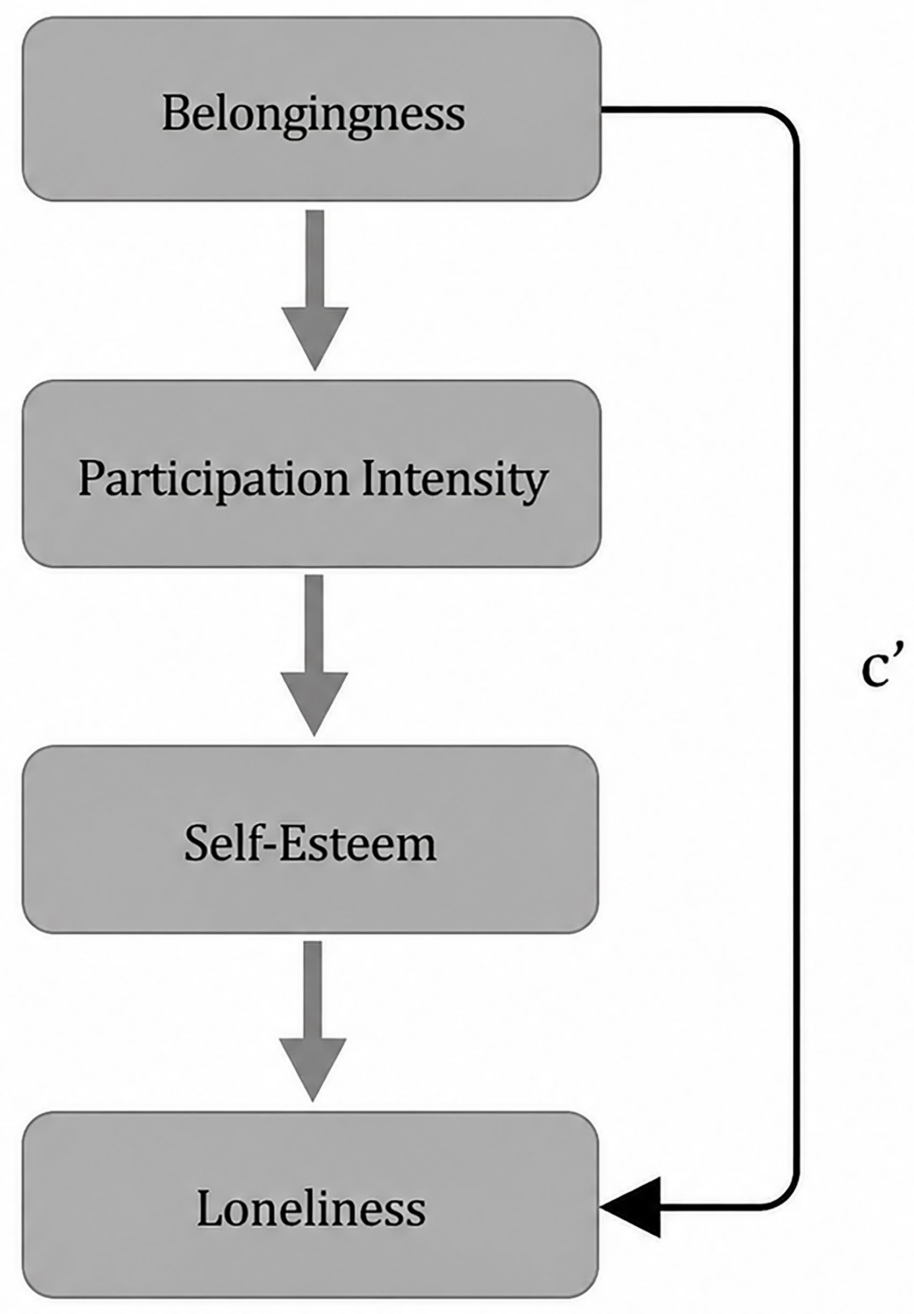
Serial mediation model linking belongingness, participation intensity, self-esteem, and loneliness.

The serial mediation model is presented in Figure 1.

The total effect of belongingness on loneliness was significant (*β* = −0.36). When the mediators were included, the direct effect remained significant (*β* = −0.23), indicating partial mediation.

Belongingness was a significant positive predictor of participation intensity (*B* = 0.52, SE = 0.04), and participation intensity significantly predicted self-esteem (*B* = 0.34, SE = 0.05). Self-esteem was a significant negative predictor of loneliness (*B* = −0.41, SE = 0.05).

Bootstrap analyses indicated that all indirect effects were statistically significant, as the 95% confidence intervals did not include zero. Specifically, the indirect effect of belongingness on loneliness through participation intensity was significant (effect = −0.054, 95% CI [−0.100, −0.011]). The indirect effect through self-esteem was also significant (effect = −0.065, 95% CI [−0.107, −0.031]). In addition, a significant serial indirect effect was observed (effect = −0.015, 95% CI [−0.030, −0.004]).

## 4 Discussion

The present study examined the relationships among belongingness in ACG communities, participation intensity, self-esteem, and loneliness among adolescents and young adults. The findings indicate that stronger perceived belongingness was associated with lower levels of loneliness, both directly and indirectly through participation intensity and self-esteem. These results extend prior research on belongingness and psychological adjustment by situating these processes within interest-based online communities.

Consistent with belongingness theory, individuals who reported a stronger sense of belonging within ACG communities tended to experience lower loneliness (Baumeister and Leary, 1995). This finding suggests that ACG communities may function as meaningful social environments rather than inherently isolating spaces. Although such communities are often portrayed as encouraging withdrawal from offline relationships, the present results indicate that perceived acceptance and connection within these communities are associated with reduced feelings of social isolation. This aligns with research showing that interest-based and online communities can provide opportunities for social connection, particularly for individuals who may feel marginalized in traditional social contexts (Kraut et al., 2014; Steinkuehler and Williams, 2006).

The mediation findings further suggest that the relationship between belongingness and loneliness is not solely direct but operates through both behavioral and intrapersonal pathways. Participation intensity emerged as an important behavioral mechanism, indicating that individuals who feel a stronger sense of belonging are more likely to engage actively in community activities. Such engagement may increase opportunities for interaction, reciprocity, and social reinforcement, thereby contributing to lower loneliness. This is consistent with prior findings that active participation, rather than passive consumption, is more strongly associated with positive psychosocial outcomes in online environments (Burke et al., 2010; Verduyn et al., 2017).

In addition, self-esteem functioned as a key intrapersonal pathway linking belongingness and loneliness. According to sociometer theory, self-esteem reflects individuals’ perceived relational value and social acceptance (Leary and Baumeister, 2000). The present findings suggest that experiences of belonging within ACG communities may enhance self-esteem, which in turn is associated with lower loneliness. Notably, the serial mediation model indicates that participation intensity precedes self-esteem in this pathway, suggesting that active engagement in community practices may provide concrete experiences of competence and recognition that are subsequently internalized into more positive self-evaluations.

Taken together, these findings contribute to the literature by integrating belongingness, participation, and self-esteem within a unified framework. Rather than viewing ACG participation primarily as a risk factor, the results highlight its potential role as a psychosocial resource. By demonstrating that belongingness within ACG communities is associated with reduced loneliness through identifiable mechanisms, the study provides a more nuanced understanding of how interest-based communities relate to youth psychological adjustment.

Several limitations should be acknowledged. First, the cross-sectional design precludes causal inference, and the directionality of the observed relationships cannot be established. Longitudinal or experimental studies are needed to clarify temporal dynamics. Second, all variables were assessed using self-report measures, which may introduce common method bias. Third, the use of convenience sampling may limit the generalizability of the findings. In addition, the study did not differentiate between online and offline forms of ACG participation, which may have distinct psychological implications. Future research could incorporate more diverse samples, multi-method assessments, and finer distinctions in participation contexts to further examine these processes.

## 5 Conclusion

The present study examined the relationships among belongingness in ACG communities, participation intensity, self-esteem, and loneliness among adolescents and young adults. The findings indicate that stronger perceived belongingness is associated with lower loneliness, both directly and indirectly through increased participation intensity and enhanced self-esteem. These results suggest that ACG communities may function as meaningful social environments that support psychological wellbeing by fostering engagement, self-worth, and social connection. By highlighting the psychological mechanisms linking belongingness to loneliness, this study contributes to a more nuanced understanding of interest-based communities in contemporary youth development.

## Data Availability

The original contributions presented in the study are included in the article/supplementary material, further inquiries can be directed to the corresponding author.
